# Pyoderma gangrenosum after radical prostatectomy: case report

**DOI:** 10.1007/s13691-018-0332-3

**Published:** 2018-06-07

**Authors:** Akiyoshi Osaka, Hisamitsu Ide, Shinichi Ban, Toshiro Takimoto, Keiichiro Aoki, Yoshitomo Kobori, Tadahiko Tokumoto, Gaku Arai, Shigehiro Soh, Yoshihiko Ueda, Hiroshi Okada

**Affiliations:** 1 Department of Urology, Saitama Medical Center, Dokkyo Medical University, 2-1-50, Minamikoshigaya, Koshigaya, Saitama 343-8555 Japan; 2 Department of Pathology, Saitama Medical Center, Dokkyo Medical University, Koshigaya, Saitama Japan

**Keywords:** Pyoderma gangrenosum, Prostate cancer, Radical prostatectomy

## Abstract

Pyoderma gangrenosum (PG) is a skin disease characterized by an unknown neutrophilic infiltration in dermis and a nonbacterial destructive ulcer. Post-operative PG is an extremely rare type that occurs around surgical sites during the immediate post-operative period. It is usually diagnosed as surgical site infection at the time of presentation. The condition rapidly worsens despite antibiotic treatment and debridement. We report on a case of post-operative PG in a 64-year-old man after radical prostatectomy. Following the operation, redness and pus from surgical site rapidly progress although repeated antibiotic therapy and debridement were performed. Although the patient received appropriate debridement and broad-spectrum antibiotic treatment, the ulcerative lesion spread surrounding drain region and the condition of the skin region deteriorated. The diagnosis of PG was made by a skin biopsy that presented only neutrophilic invasion in the dermis without vasculitis, tumor, or malignancy. Finally, the patient died of lesion progression in whole body and multiple organ dysfunction. Considering PG along with ulcers, wounds, and post-operative complications is critical for prompt diagnosis and proper treatment.

## Introduction

Pyoderma gangrenosum (PG) is frequently associated with systemic disease and is often confused with other skin pathergies [1, 2]. PG occurs in approximately three cases in a population of one million individuals, with over half of all cases associated with immune system disorders in an underlying systemic disease. The condition develops rapidly and forms deep ulcers. Post-operative PG is extremely rare and typically develops around surgical sites within the first 2 weeks post-operatively [3]. Therefore, it is often misdiagnosed as wound infection, and the pathergy may complicate wound debridement with rapid ulcer development [4]. This condition has clinical features analogous to infectious processes. The following report describes a case of post-operative PG in a 64-year-old man after retropubic radical prostatectomy.

## Case report

**Fig. 1:**
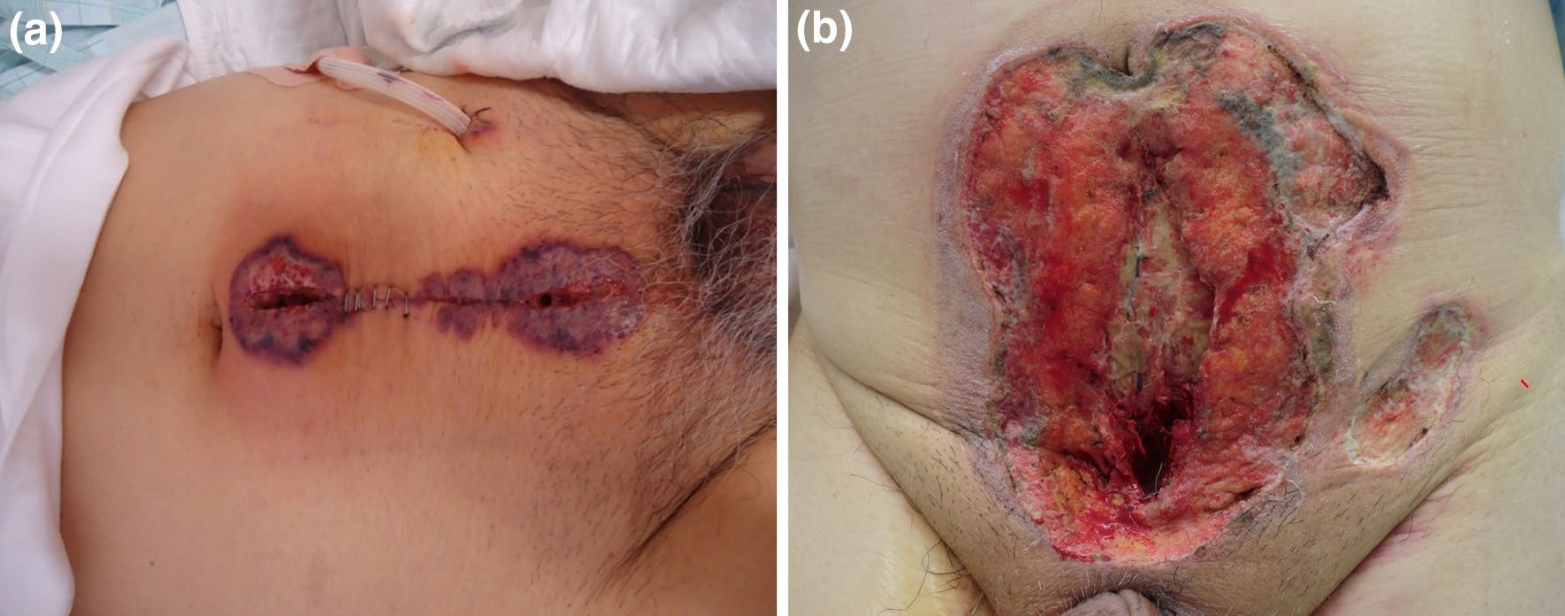
Post-operative pyoderma gangrenosum. Erythema occurred in the surgical site 4 days after radical prostatectomy (**a**). The skin inflammation change spread with deep erosion despite introduction of debridement. Inflammation continued to spread even 15 days following surgery (**b**)

**Fig. 2:**
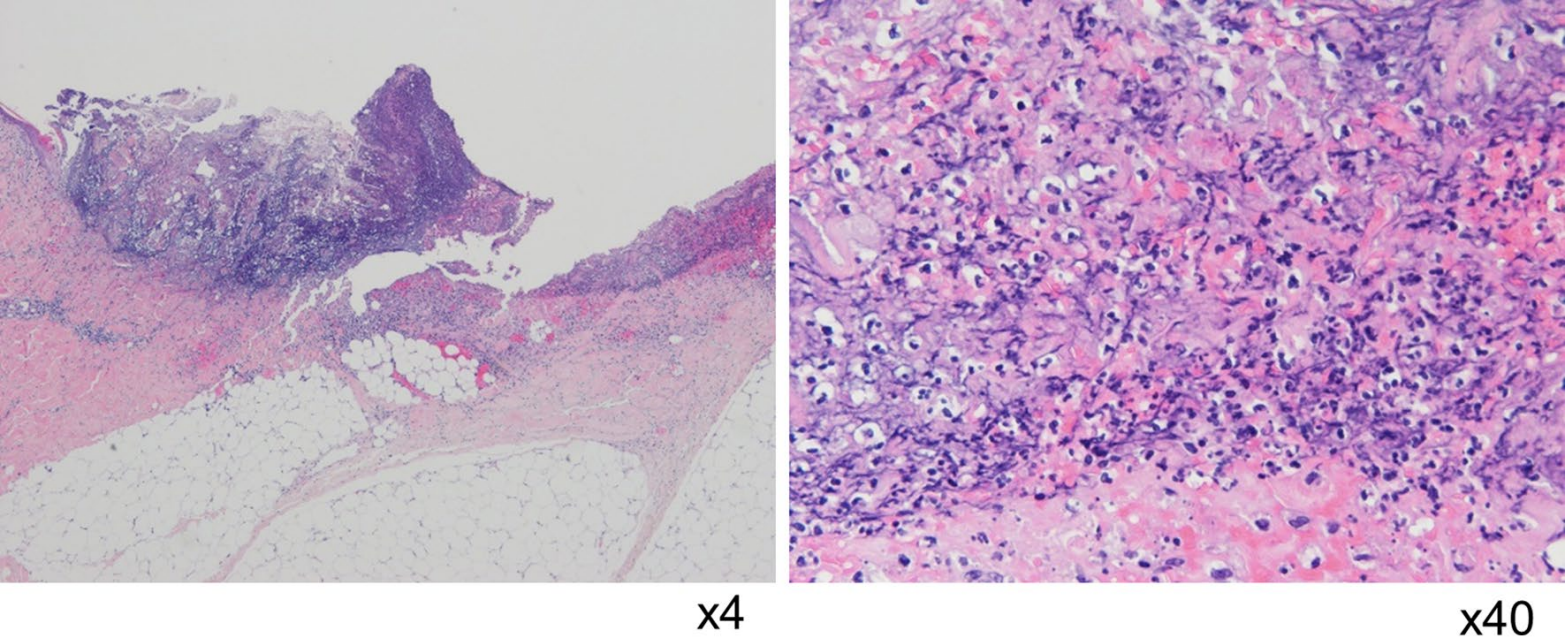
Histological findings of the skin biopsy of post-operative pyoderma gangrenosum. Pathological features revealed that severe inflammatory cells, predominantly of neutrophils, infiltrated in the dermis. No bacterial components or tumor cells were observed. Hematoxylin–eosin stain ×4 (**a**), ×40 (**b**)

A 64-year-old man with a history of hypertension was referred to our hospital with a high serum level of prostate-specific antigen (9.01 ng/ml). The patient had no medical history of immune disease such as inflammatory disease, arthritis or hematological disease. The result of a systemic prostate biopsy showed prostate cancer with a Gleason score of 7, and a clinical stage of cT2bN0M0. The patient underwent a retropubic radical prostatectomy. We treated the patient with ampicillin sulbactam for prevention of post-operative infection. The site of the surgical wound and drain insertion presented redness and produced pus in addition to prolonged fever for 4 days following surgery. Following the diagnosis of surgical site infection, antimicrobial therapy using meropenem that is a broad-spectrum antibacterial agent of the carbapenem family was initiated (Fig. 1a). Although the patient received appropriate debridement and broad-spectrum antibiotic treatment, the ulcerative lesion spread surrounding drain region and the condition of the skin region deteriorated 10 days following surgery. The patient presented kidney and liver dysfunction and was transferred to the Intensive Care Unit. Despite treatment with an additional antifungal agent and debridement, there was no improvement (Fig. 1b). Blood, urine, sputum and wound culture were negative for any pathogen. The diagnosis of PG was made by a skin biopsy that presented only neutrophilic invasion in the dermis without vasculitis, tumor, or malignancy 37 days following surgery (Fig. 2). Although treatment with 80 mg/day intravenous prednisolone was initiated, the patient died of multiple organic dysfunction due to liver, heart, and kidney dysfunction.

## Discussion

Brunsting et al. [5] initially described PG as an uncommon inflammatory ulcerative skin disease. It typically occurs in the lower extremities at any age. Although approximately 50% of all cases are associated with autoimmune diseases such as inflammatory bowel disease, rheumatoid arthritis and hematological malignancy, PG is difficult to differentiate from similar etiologies, including infectious, vasculitic, drug-induced and other inflammatory dermatoses [6–8]. The pathophysiology of post-operative PG in particular has remained unknown, but the primary cause could be polymorphonuclear neutrophils chemotaxis following post-operative upregulation of cytokine release [9–11] Most cases of post-operative PG follow breast surgery or, occasionally, abdominal, gynecological or heart surgery [3]. To our knowledge, the present case was first report of post-operative PG after urological surgery.

In the present case, the other differential diagnosis we must considered was drug-induced PG [8]. The Naranjo score for determining the likelihood of whether an adverse drug reaction is actually due to the drug rather than the result of other factors. The score is one for indicating a possibility of adverse drug reaction was extremely low.

As diagnosis of PG is challenging due to the absence of laboratory findings and pathology, a definite diagnosis is determined by clinical course, ulcerative lesion, failed responsiveness to antibiotic treatment and debridement, and a skin biopsy ruling out other disease. Unfortunately, post-operative PG has often been misdiagnosed as severe surgical site infection due to similar clinical presentation. In fact, a retrospective study of 240 cases showed misdiagnosis in 95 cases [12]. Delayed diagnosis leads to deteriorating ulcerative lesions by repeated debridement and an increased mortality rate [13]. However, early intervention with steroids produces great improvement within 24 h and a reduced mortality rate that generally estimated up to 22.4% [13, 14].

Brown et al. suggested that PG was systemic disease and extracutaneous involvement might exist at the present of skin lesions [15]. 80% of the PG patients who died had an associated systemic disease, although we could not diagnose definite systematic diseases in our case. In addition, our case may have a possible correlation between infected PG wounds and poorer prognosis which has been reported in the literature [16].

As one of the main causes of pathergy was surgery, we need to consider if surgical site is worsening despite repeated debridement and broad antibiotic therapy. Although there are currently no uniformly accepted diagnostic criteria for PG, this disease is characterized by the appearance of a painful, irregular ulcer with a violaceous border. It is critical for clinicians to be aware and vigilant in diagnosing this complication as delayed diagnosis can potentially lead to poorer prognosis as our case.
